# P35 Impact of IV to oral antibiotic switch optimization on nurse time and plastic waste at the Royal Cornwall Hospital Trust

**DOI:** 10.1093/jacamr/dlae136.039

**Published:** 2024-08-23

**Authors:** Michelle Tan, Neil Powell, Daniel Hearsey

**Affiliations:** Pharmacy Department, Treliske Hospital, Royal Cornwall Hospitals NHS Foundation Trust, UK; Pharmacy Department, Treliske Hospital, Royal Cornwall Hospitals NHS Foundation Trust, UK; Pharmacy Department, Treliske Hospital, Royal Cornwall Hospitals NHS Foundation Trust, UK

## Abstract

**Background:**

IV to oral switch (IVOS) is an important antimicrobial stewardship intervention with benefits that include a lower risk of bloodstream and catheter-related infections, reduced equipment costs, lower carbon footprint and reduced hospital length-of-stay as well as releasing nursing time to care for patients.^1–3^ We quantified the impact of more prompt IVOS on nurse drug administration time and unrecyclable waste generated from IV antibiotic administration.

**Methods:**

Data were collected during three lunchtime drug administration rounds each week on the Acute Medical Unit (AMU) and the Emergency Surgical Unit (ESU) over a 4 week period in December 2023. A trainee pharmacist timed how long it took nurses to make up and administer both IV and oral antibiotic doses. Data collected included: antibiotic name, antibiotic route, IV antibiotic administration method (if applicable), time taken for drug preparation, time taken for preparation and to set up for administration. Photographs of the waste generated were obtained for each antibiotic. To estimate the potential for reducing excess IV doses, and to estimate potential nurse time savings, we used the Trust’s IVOS CQUIN data to estimate excess IV antibiotic use and EPMA IV administration data to quantify the number of IV antibiotic doses administered during the study period.

**Results:**

The preparation and administration of 56 antibiotics (34 IVs and 22 orals) was observed for 53 patients. The mean time to prepare and administer IV infusions was 10:27 min and 5:53 min for IV bolus injections. Oral antibiotics took 2:42 min. During the audited month, a total of 24 248 doses of IV antibiotics were administered across all inpatient wards at RCHT, which equates to 782 doses per day. According to our data, 782 doses of IV antibiotics administration would require approximately 136 h of nursing time every single day. Based on our IVOS CQUIN data, approximately 11% (86 doses) of IV antibiotics could be switched to oral. This equates to 86 doses of IV antibiotics which could be saved and changed to oral which could save approximately 12 h of nursing time every day.

**Conclusions:**

Optimizing IV to oral switching could save 12 h of nursing time every day. In addition, we could reduce our carbon footprint by reducing the generation of non-reusable plastic produced in the process of administering IV antibiotics compared with oral antibiotics.
